# Functional magnetic resonance imaging reveals differences in brain activation in response to thermal stimuli in diabetic patients with and without diabetic peripheral neuropathy

**DOI:** 10.1371/journal.pone.0190699

**Published:** 2018-01-05

**Authors:** Juan Li, Wanying Zhang, Xia Wang, Tangmi Yuan, Peiyao Liu, Tao Wang, Le Shen, Yuguang Huang, Naishi Li, Hui You, Tixian Xiao, Feng Feng, Chao Ma

**Affiliations:** 1 Department of Anesthesiology, Peking Union Medical College Hospital, Beijing, China; 2 Department of Radiology, Peking Union Medical College Hospital, Beijing, China; 3 Department of Anatomy, Histology and Embryology, Institute of Basic Medical Sciences Chinese Academy of Medical Sciences, School of Basic Medicine Peking Union Medical College, Beijing, China; 4 State Key Laboratory of Medical Molecular Biology & Department of Immunology, Institute of Basic Medical Sciences, Chinese Academy of Medical Sciences, Beijing, China; 5 Department of Radiation Oncology, Fudan University Shanghai Cancer Center, Shanghai, China; 6 Department of Endocrinology, Peking Union Medical College Hospital, Beijing, China; 7 Medical College of Soochow University, Suzhou, Jiangsu, China; 8 Neuroscience Center, Chinese Academy of Medical Sciences, Beijing, China; Weill Cornell Medical College in Qatar, QATAR

## Abstract

**Introduction:**

Diabetes affects both the peripheral and central nervous systems. The aim of this study was to explore the changes in brain activity in response to thermal stimuli in diabetic patients with and without diabetic peripheral neuropathy (DPN) using functional magnetic resonance imaging (fMRI).

**Methods:**

A total of 36 right-handed volunteers were enrolled: eight patients with Type-2 diabetes mellitus and DPN, 13 patients with Type-2 diabetes mellitus lacking DPN (NDPN patients), and 15 healthy volunteers (HV). Blood oxygenation level-dependent baseline scans were performed, first without any stimuli, and then with four sessions of thermal stimuli (0, 10, 34, and 44°C, in a random order) applied to the lateral side of the right lower extremity. There was a 240-s rest interval between each thermal stimulation. Each stimulation session consisted of three cycles of 30 s of stimulation followed by 30 s of rest. After each stimuli session, the participant rated pain and itch perception on a visual analog scale. The fMRI data series were analyzed by using Statistical Parametric Mapping 8 and Data Processing Assistant for Resting-State fMRI.

**Results:**

In response to temperature stimuli, DPN patients showed stronger activation than HV and NDPN patients, not only in brain areas that participate in somatosensory pathways (right insula, left caudate nucleus, frontal gyrus, and cingulate cortex), but also in the cognition-related cerebral areas (right temporal lobe, left hippocampus, and left fusiform gyrus). Activation of vermis 1–3 was greater in NDPN patients than in HV in response to 0°C stimulation.

**Conclusions:**

fMRI may be useful for the early detection of central nervous system impairment caused by DPN. Our results indicate that central nervous system impairment related to diabetic neuropathy may not be limited to motion- and sensation-related cortical regions. Cognition-associated cerebral regions such as the hippocampus and fusiform gyrus are also affected by functional changes caused by DPN. This suggests that fMRI can detect the early stages of cognitive impairment in DPN patients before the symptoms become clinically significant.

## Introduction

Diabetic peripheral neuropathy (DPN), a very common late complication of diabetes mellitus (DM), affects up to 50% of patients with the condition and causes progressive disability [1]. DPN is a symmetrical, length-dependent sensorimotor polyneuropathy caused by metabolic changes and microvascular alterations resulting from exposure to hyperglycemia and other risk factors [2]. DPN may present with features varying from autonomic impairment to sensory deficit. Pain is present in 16–21% of patients [3]. The gain-of-function mutation of sodium channel Nav1.7, which leads to small-fiber neuropathy, is reported to raise susceptibility to neuropathic pain [4]. The gold-standard method of diagnosing DPN is electromyography (EMG). However, many DPN patients do not have neuropathic symptoms despite showing evidence of defective nerve function on EMG. Due to the insidiousness of onset of symptoms, DPN is often not diagnosed in the early stages.

Over the past few decades, neuroimaging has been applied to the study and early detection of DPN, and has demonstrated functional changes caused by DPN in the central nervous system (CNS) [5]. Molecular imaging modalities such as positron emission tomography, single-photon emission computed tomography, and magnetic resonance spectroscopy have been used to examine brain functional changes in DPN patients. However, these modalities are not suited to routine clinical use because of their lengthy scanning time and high cost.

Functional magnetic resonance imaging (fMRI) is noninvasive and radiation-free, and can be used multiple times in the same patient or to scan large samples. Most importantly, it directly relates real-time functional changes in specific central nuclei and cortical regions to external stimuli. fMRI capitalizes on the differences in magnetic effects between oxygenated and deoxygenated blood, which exactly reflect the local oxygen consumption of regions of the CNS due to external stimuli [6]. This blood oxygenation level-dependent (BOLD) imaging can demonstrate brain activation in response to different stimuli [6], improving our understanding of how DPN and the CNS are interrelated.

fMRI is becoming a new and practical tool in DPN imaging. Some studies have reported the utility of fMRI in examining brain activity in DPN patients, and demonstrated significant activation of the primary sensory cortex, frontal lobe, thalamus, and cerebellum in DPN patients [7–9]. However, most of these studies have focused on the resting state (without external stimulation) and reported widely divergent results [10]. We hypothesized that DPN patients may have different CNS functional changes (as indicated by their response to thermal stimuli) to DM patients without DPN (NDPN patients), which may reflect early CNS impairment in DPN patients. To prove this, we examined the responses of the cerebral cortex and nuclei to different temperature stimuli (0, 10, 34, and 44°C) in DPN patients, NDPN patients, and healthy volunteers (HV).

## Materials and methods

### Participants

The research protocol was approved by the Institutional Review Board of the Institute of Basic Medical Sciences of the Chinese Academy of Medical Sciences, Peking Union Medical College, Beijing, China (Approval Number: 011–2014). Informed consent was obtained from all participants.

A total of 36 right-handed volunteers aged 45–65 years were enrolled into three groups in this study: 1) a DPN group, comprising eight patients with Type-2 DM and DPN—these patients had experienced bilateral painless sensory symptoms (confirmed by EMG) involving the feet for over 6 months; 2) an NDPN group, comprising 13 patients with Type-2 DM patients lacking DPN; and 3) an HV group, comprising 15 healthy individuals. Patients were excluded from the study if: 1) T2-weighted MRI revealed cerebral infarct, hemorrhage, or subcortical arteriosclerotic encephalopathy; 2) antihistamine drugs or anti-pain medication had been taken less than 24 hours before the start of the experiment; 3) there were complaints of pain elsewhere in the body (e.g., headache, backache, or stomach pain); 4) there was any contraindication for MRI (e.g., defibrillator, cardiac pacemaker, or insulin pump use); and 5) psychiatric examination (Mini-Mental State Examination [MMSE]) revealed any disorder. Cognitive impairment was defined as an MMSE score lower than 27.

### Thermal stimulation

The stimulation site was at the lateral side of the right lower extremity, about 25 cm above the lateral malleolus. The heat stimulus was applied using a 200-mL beaker that was immersed in 1000 mL of water at different temperatures (0, 10, 34, and 44°C) before application. The beaker was lightly applied to the marked site without exerting any pressure; the contact area was approximately 3 cm^2^. The temperature of the water in the beaker was measured before and after application. The participants received thermal stimuli at four different temperatures in a random order.

### Experimental protocol

**Fig 1:**
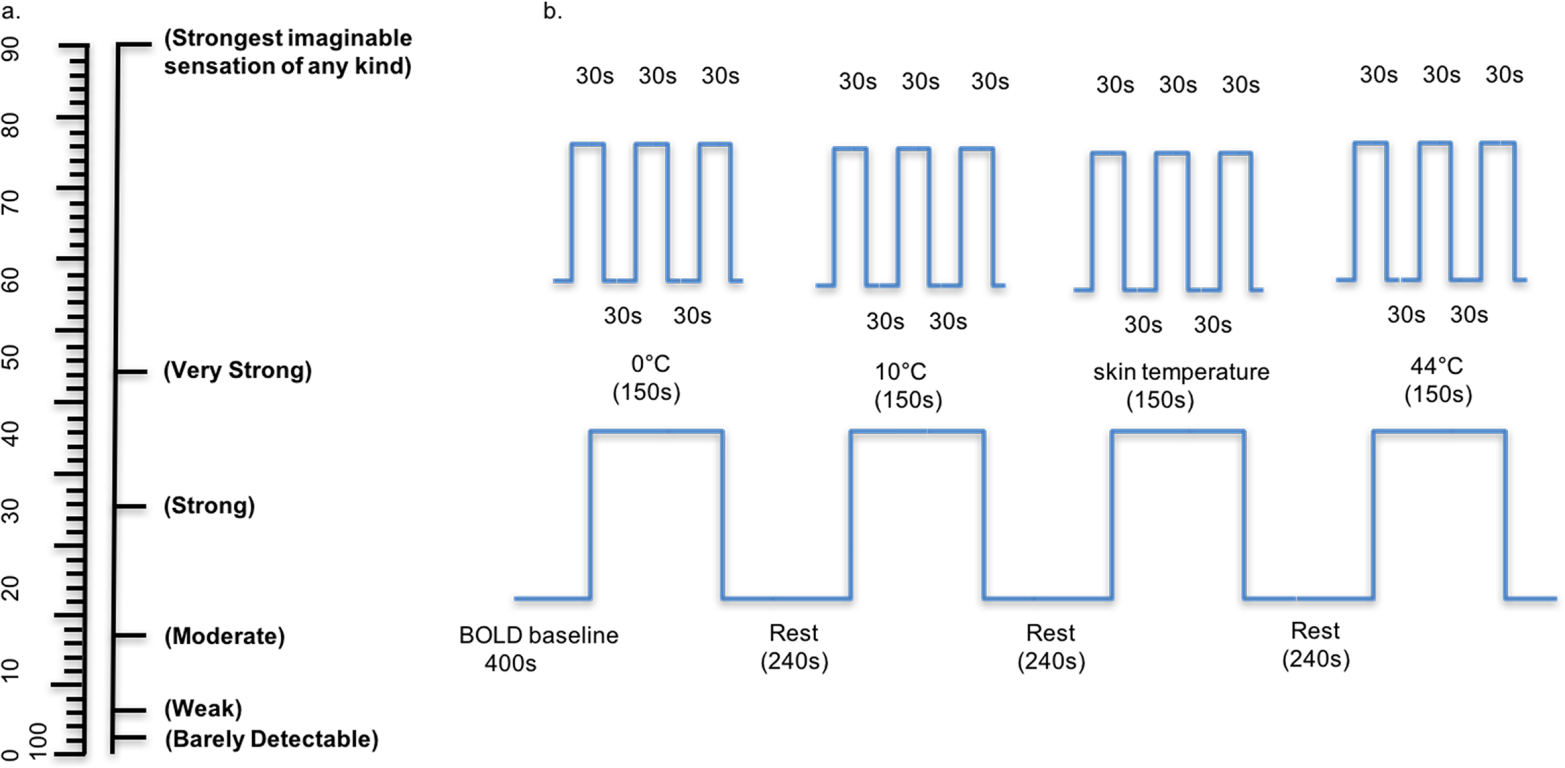
Visual analog scores and the functional magnetic resonance imaging scanning workflow. (a) The visual analog scale. (b) The functional magnetic resonance imaging scanning workflow. Each participant first underwent a 400-s baseline scan; then, scans were performed during stimulus sessions (0°C→10°C→34°C→44°C in a random order), with a 240-s rest interval between each session. To prevent stimulation desensitization, each stimulus session included three cycles of 30 s of stimulation and 30 s of rest.

Following stimulation, the participants first classified the perceived sensation as an “itching sensation” (desire to scratch), “tingling sensation” (sharp and accurately positioned, intermittent or constant painful sensation), or “burning sensation” (similar to that felt on exposure to strong sunshine, sometimes along with skin rupture and chemical stimuli). Next, the strength of the perceived sensation was indicated on a visual analog score (VAS) scale (Fig 1A) on which a score of 0 indicated “no sensation”; 1 indicated “barely detectable” sensation; 5 indicated “weak” sensation; 15 indicated “moderate” sensation; 35 indicated “strong” sensation (i.e., causing discomfort but still bearable); 50 indicated “very strong” sensation (cannot help scratching or attempting to get rid of stimuli); and 100 indicated “strongest imaginable” sensation.

Participants were requested to not take any anti-neuropathy drugs on the day of the experiment and to abstain from stimulating agents such as coffee or tea for 6 hours prior to the test. The Michigan Neuropathy Screening Instrument (MNSI) was used to screen and type the clinical neuropathy of patients in the DPN and NDPN groups [11,12]. The EMG results demonstrated that the DPN patients had peripheral neuropathy of the bilateral lower extremities, confirming the diagnosis of DPN. No EMG tests were performed on NDPN patients.

### Image acquisition

A 3-T MRI scanner (GE Discovery MR750 3.0T) was used for fMRI. The same experienced technician performed the examination in all participants. The participant’s head was comfortably positioned inside an eight-channel head coil and fixed with a strap across the forehead. Ear plugs were used to minimize scanning noise. The participant was asked to keep their eyes closed and mind clear during the scan. A T1-weighted anatomical image was first collected using 3D fast spoiled gradient echo (3D FSPGR) imaging, with parameters as follows: echo time (TE) 3.4 ms, recovery time (TR) 8.6 ms, FS 3, slice thickness 1.1 mm, slice gap 0 mm. BOLD data were collected using the following parameters: TE 30.0 ms, TR 2000.0 ms, FS 3, slice thickness 4 mm, and slice gap 0 mm.

A 400-ms BOLD baseline scan (without any stimulus) was first performed; then, scans were performed during the application of random thermal stimuli. For example, following the baseline scan, the participant shown in Fig 1B was scanned during a thermal stimulus session consisting of 0°C→10°C→34°C→44°C (or in a random order), with a 240-s rest interval between each session. To prevent stimulation desensitization, each stimulation session consisted of three cycles of 30 s of stimulation followed by 30 s of rest. After each functional scan, the participant verbally rated the overall perception (pain or itch) and the strength of the stimulus on the VAS.

### Functional magnetic resonance imaging data analysis

#### Preprocessing

As fMRI signals are often corrupted by random noise, it is important to have these components appropriately modeled. fMRI data typically undergo preprocessing to remove physiological artifacts and validate model assumptions. The major steps involve motion correction, realignment, coregistration, normalization, and smoothing.

In this study, participants with a head movement exceeding 2 mm of maximum translation in any of the *x*, *y*, or *z* axes or 2^o^ of maximum rotation about the three axes were excluded from this study to minimize movement artifacts. The fMRI sequence was interleaved. A total of 76 volumes was scanned for each individual, and the first volume was discarded to allow for signal equilibrium of the initial MRI signals and adaptation of the participants to the circumstances. The remaining 75 consecutive volumes were used for data analysis. The fMRI data series were analyzed using Statistical Parametric Mapping 8 (SPM8) and Data Processing Assistant for Resting-State fMRI as described previously [13–15]. Briefly, the data series were aligned to the first image in each scan sequence and resampled by interpolation to correct voxel values, thus correcting motion artifacts. This procedure was repeated for each individual. Next, the functional data were coregistered to the T1-weighted anatomical image. Each participant’s anatomy was registered to a template brain provided by the Montreal Neurological Institute. Mapping was used to wrap the input image on to the template image, to produce a normalized image that could be compared between participants. The resampled voxel volume of the normalized images was 3-mm isotropic. Subsequently, smoothing was performed using a Gaussian kernel with a 4-mm full width at half maximum to increase the signal-to-noise ratio within the region. Conditional specific effects were estimated using the general linear model (GLM) in SPM8, which was constructed by convolving a boxcar sequence with the hemodynamic response function. A high-pass filter with a cutoff period of 128 s was used to remove low-frequency noise.

After the above preprocessing steps, the data series satisfied Gaussian distribution and were subjected to statistical analysis. In addition, slice timing correction was also performed [9,16].

#### Data analysis

Interaction analysis was used to compare brain activities between the DPN, NDPN, and HV groups.

SPM8 was applied to compare the brain activation pattern in the three groups to establish its relationship with stimulation (cluster size k > 20; *p* < 0.05). One-way analysis of covariance with a GLM was used to analyze the interaction effect and bivariate covariance, with age and sex treated as covariant factors, followed by *post-hoc* analysis for differences between conditions. T0_task, T10_task, T34_task, and T44_task represented the fMRI data achieved under 0, 10, 34 (skin temperature), and 44°C stimulation. T0_off, T10_off, T34_off, T44_off represented the taking-off of these stimuli. A 3 × 3 bivariate covariance model was established with factor 1 set as DPN, NDPN, and HV, factor 2 set as (T0_task − T0_off) − (T34_task − T0_off), (T10_task − T10_off) − (T34_task − T0_off), (T44_task − T44_off) − (T34_task − T0_off) and change in skin temperature in response to the stimuli. All multiple comparisons were uncorrected, but set at a threshold of *p* < 0.05 and a cluster size of > 20 voxels.

The demographic features of the three groups were analyzed using different methods based on their distribution. Age, education, and body mass index (BMI) were analyzed using analysis of variance (ANOVA). The duration of DM, DPN, hypertension, and dyslipidemia, as well as the hours of exercise per day, were analyzed using a nonparametric test because of their non-normal distribution. The relationship between smoking history, alcohol consumption, and DM were tested using a likelihood ratio chi-squared test. MNSI score was compared between the DPN and NDPN groups using the *t* test. VAS score was evaluated by ANOVA. Statistical significance was set at *p* < 0.05. All analyses were performed using SPSS 17.0 software (SPSS Inc., Chicago, IL, USA).

## Results

### Demographics

A total of 36 right-handed volunteers were enrolled: eight in the DPN group, 13 in the NDPN group, and 15 in the HV group. The DPN patients had significantly higher MNSI values, at 8.75 ± 1, than the NDPN patients, at 0.38 ± 0.51 (p < 0.001). The EMG results demonstrated that DPN patients had peripheral neuropathy of the bilateral lower extremities, confirming their diagnoses of DPN. No EMG tests were performed on NDPN patients. The results of these analyses are shown in Table 1.

**Table 1:** Michigan Neuropathy Screening Instrument and electromyography results for diabetic patients with and without diabetic peripheral neuropathy.

**DPN**	**MNSI**	**EMG**
1	7.5	Bilateral lower extremity peripheral neuropathy
2	8	Bilateral lower extremity peripheral neuropathy
3	8	Bilateral lower extremity peripheral neuropathy
4	10	Bilateral lower extremity peripheral neuropathy
5	9.5	Bilateral lower extremity peripheral neuropathy
6	9	Bilateral lower extremity peripheral neuropathy
7	10	Bilateral lower extremity peripheral neuropathy
8	8	Bilateral lower extremity peripheral neuropathy
Mean ± SD	8.75 ± 1	
**NDPN**	**MNSI**	**EMG**
1	1	No EMG tested
2	0	No EMG tested
3	0.5	No EMG tested
4	0	No EMG tested
5	1	No EMG tested
6	0.5	No EMG tested
7	0	No EMG tested
8	1.5	No EMG tested
9	0	No EMG tested
10	0	No EMG tested
11	0	No EMG tested
12	0.5	No EMG tested
13	0	No EMG tested
Mean ± SD	0.38 ± 0.51	

The mean ± standard deviation Michigan Neuropathy Screening Instrument scores of patients with and without diabetic peripheral neuropathy were 8.75 ± 1 and 0.38 ± 0.51, respectively (*t* test, *p* < 0.001).

DPN, diabetic peripheral neuropathy; MNSI, Michigan Neuropathy Screening Instrument; EMG, electromyography; SD, standard deviation; NDPN, diabetes mellitus without diabetic peripheral neuropathy.

There was no significant difference between the three groups in terms of mean age (*p* = 0.962), sex distribution (*p* = 0.769), BMI (*p* = 0.612), prevalence of hypertension (*p* = 0.627) and dyslipidemia (*p* = 0.45), mean hours of exercise (*p* = 0.096), and the proportions of smokers (*p* = 0.076) or alcohol users (*p* = 0.427; Table 2).

**Table 2:** Demographic characteristics of healthy volunteers and diabetic patients with and without diabetic peripheral neuropathy.

	HV	NDPN	DPN	Total	*p*
Number of participants	15	13	8	36	
Sex (M/F)	6/9	7/6	4/4	17/19	0.769
Age (years) ^a^	56.1 ± 7.4	55.8 ± 11.9	55 ± 7.9	55.8 ± 9.1	0.962
Education (years) ^a^	13 ± 3	12.5 ± 2.5	13.7 ± 4	12.9 ± 2.8	0.074
BMI (kg/m^2^)^a^	26.5 ± 2.9	26 ± 3.7	24.2 ± 4.5	26 ± 3.4	0.612
DM duration (years)^b^	0 (0–0)	4.5 (0–13)	12.5 (3–30)	2 (0–30)	< 0.001
DPN duration (years)^b^	0 (0–0)	0 (0–0)	3 (1–27)	0 (0–27)	< 0.001
Hypertension duration (years)^b^	0 (0–20)	0 (0–10)	5 (0–10)	0 (0–20)	0.627
Dyslipidemia duration (years)^b^	0 (0–5)	0 (0–10)	0 (0–10)	0 (0–10)	0.45
Exercise (hours per day)^b^	1 (1–2)	1.5 (1–3)	0.5 (0.5–1)	1 (1–3)	0.096
Proportion of smokers (%)	7/30 (23.2%)	11/26 (42.3%)	9/16 (56.3%)	27/72 (37.5%)	0.076
Proportion of alcohol users (%)	5/20 (25%)	7/26 (26.9%)	7/16 (43.8%)	19/72 (26.4%)	0.427

^a^Age, education, and body mass index were expressed as means ± standard deviation and analyzed using analysis of variance.

^b^The durations of diabetes, diabetic peripheral neuropathy (DPN), hypertension, and dyslipidemia, and the hours of exercise per day, were expressed as medians (ranges) and analyzed using a nonparametric test. The proportion of smokers in each group was estimated from the average of the proportion of smokers assuming all those lost to follow-up didn’t smoke and the proportion of smokers assuming all those lost to follow-up smoked. The relationships between smoking history and diabetes and alcohol consumption and diabetes were tested using a likelihood ratio chi-squared test. All the patients with or without DPN were diagnosed with Type-2 diabetes mellitus. *p* < 0.05 was considered statistically significant.

HV, healthy volunteer; NDPN, diabetes mellitus without diabetic peripheral neuropathy; DPN, diabetic peripheral neuropathy; BMI, body mass index; DM, diabetes mellitus.

### Brain activities in response to different temperature stimuli

**Fig 2:**
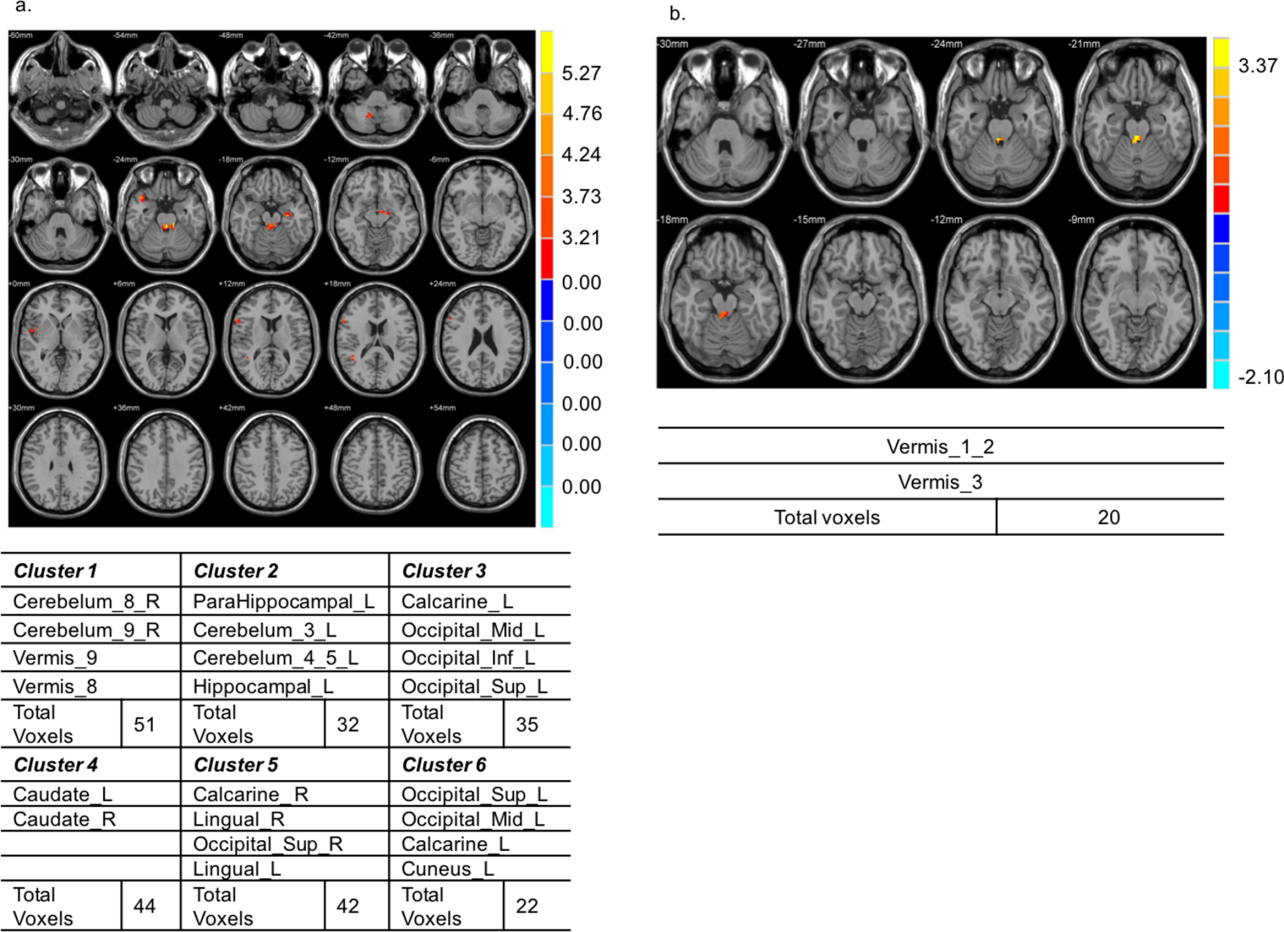
Comparison of brain activities during the general interaction analysis and in response to 0°C stimulation between diabetic patients lacking diabetic peripheral neuropathy and healthy volunteers. (a) The cerebellum, vermis, hippocampus, calcarine fissure, occipital cortex, and caudate nucleus were activated in diabetic patients lacking diabetic peripheral neuropathy (NDPN patients). (b) Brain activity in the vermis 1–3 in response to 0°C stimulation was significantly higher in NDPN patients.

Fig 2A shows that the cerebellum, vermis, hippocampus, calcarine fissure, occipital cortex, and caudate nucleus were activated in NDPN patients. However, when using BOLD in the resting state (instead of skin temperature) as the baseline, the fMRI data could not be corrected and the corresponding brain activity had no statistical significance. We therefore chose skin temperature as the baseline.

**Fig 3:**
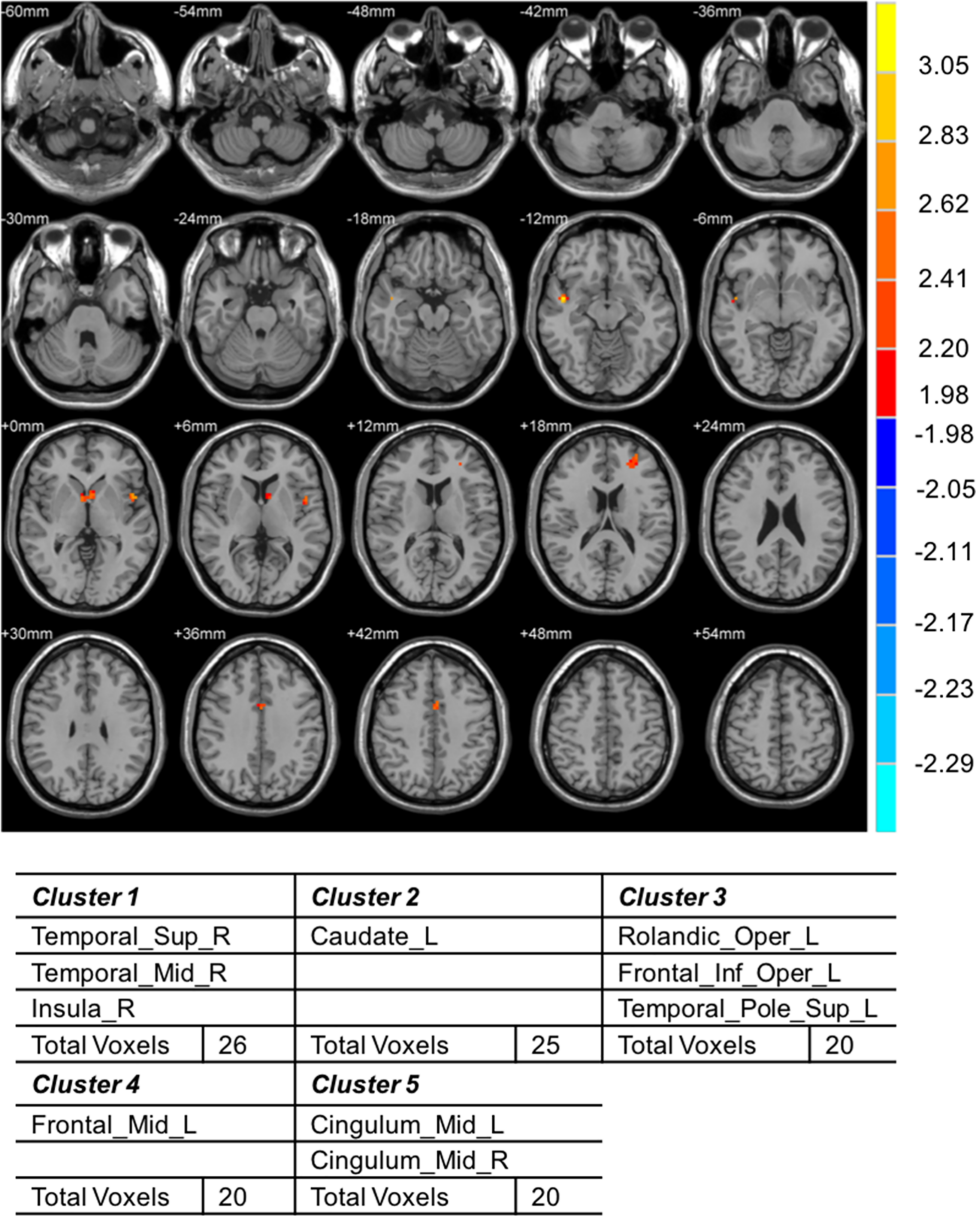
Comparison of brain activities during the general interaction analysis between diabetic patients with diabetic peripheral neuropathy and healthy volunteers. The temporal lobe, right insular cortex, left caudate nucleus, left rolandic operculum, frontal gyrus, and cingulate cortex were significantly activated in diabetic patients with diabetic peripheral neuropathy compared with in healthy volunteers.

**Fig 4:**
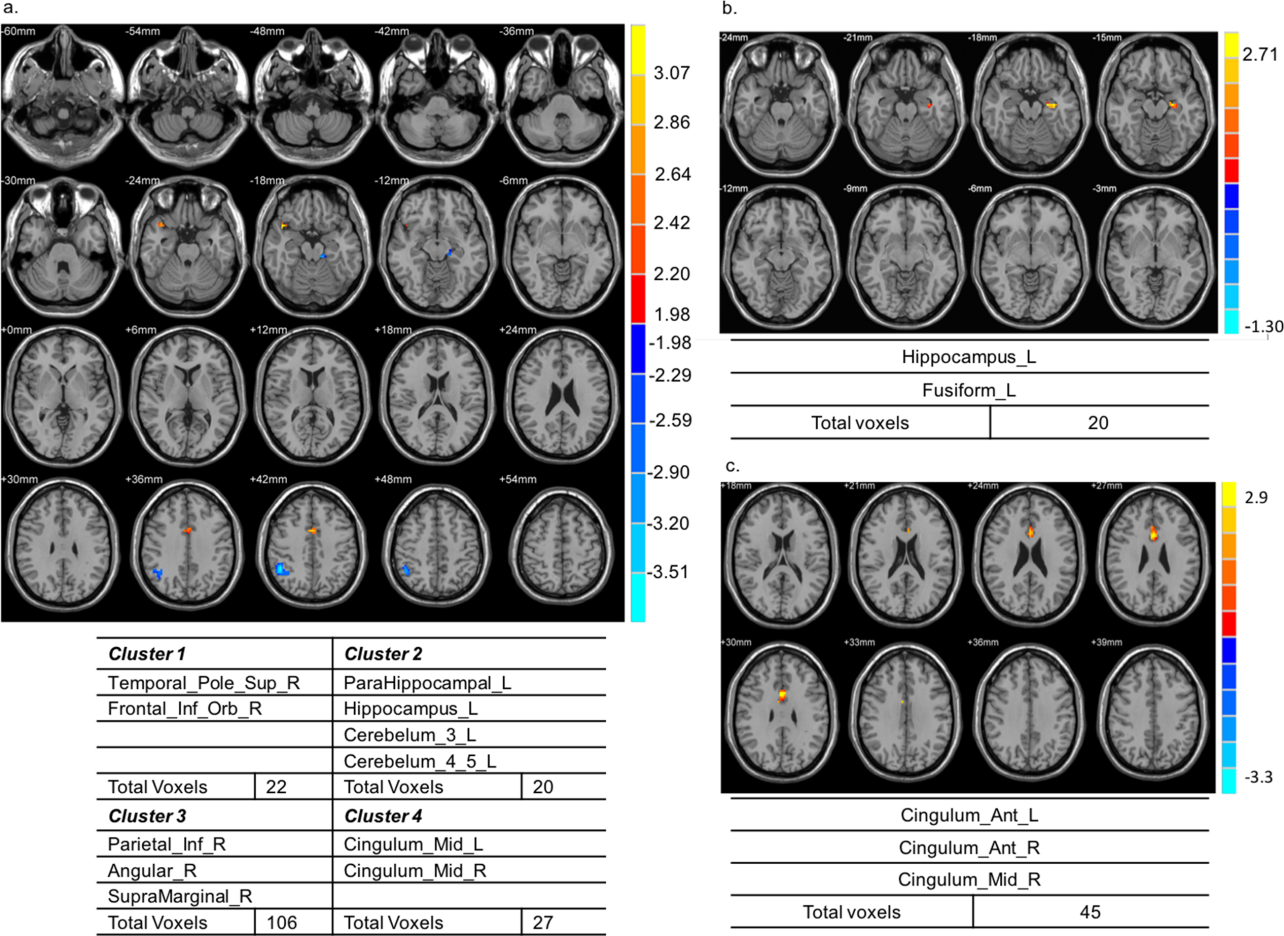
Comparison of brain activities during the general interaction analysis and in response to specific thermal stimulation between diabetic patients with and without diabetic peripheral neuropathy. (a) The temporal pole, hippocampus, cerebellum, right angular gyrus, right supramarginal gyrus, and bilateral cingulum were significantly activated in diabetic patients with diabetic peripheral neuropathy. (b) The left hippocampus and left fusiform gyrus were activated in response to 10°C stimulation. (c) The left anterior, right anterior, and right middle cingulum were activated in response to 44°C stimulation.

The three groups had distinct brain activation patterns in response to both general interaction analysis and specific thermal stimuli. When the conditions were set at *p* < 0.05 and cluster size k > 20 with skin temperature as the baseline, the general interaction analysis between DPN patients and HV indicated that the following clusters were significantly activated: the temporal lobe, right insular cortex, left caudate nucleus, left rolandic operculum, frontal gyrus, superior temporal pole, and cingulate cortex (Fig 3). However, for each thermal stimulation, the patterns were not significantly different between the groups. Similarly, in the general interaction analysis between NDPN patients and HV, the cerebellum, vermis, hippocampus, calcarine fissure, occipital cortex, and caudate nucleus were activated in NDPN patients (Fig 2A). Moreover, the brain activity level in vermis 1–3 in response to 0°C stimulation was significantly higher in NDPN patients than in HV (Fig 2B). The fMRI activation pattern partially agreed with the VAS results regarding the increase in VAS results in NDPN patients, although without statistical significance (Table 3). Moreover, the general interaction analysis between DPN and NDPN patients showed statistically significant activation in the temporal pole, hippocampus, cerebellum, right angular gyrus, right supramarginal gyrus, and bilateral cingulum in DPN patients (Fig 4A). The differences were also significant in response to 10 and 44°C stimulation. In DPN patients, the left hippocampus and left fusiform gyrus were activated in response to 10°C stimulation (Fig 4B), whereas the left anterior, right anterior, and right middle cingulum were activated in response to 44°C stimulation (Fig 4C).

**Table 3:** Visual analog scores in healthy volunteers and diabetic patients with and without diabetic peripheral neuropathy.

	Thermal stimuli (°C)	VAS			ANOVA *p* value
		HV	NDPN	DPN	
**Pain**	**0**	5 ± 9.45	6.15 ± 9.64	1.25 ± 3.53	0.445
	**10**	4 ± 7.12	5.31 ± 8.04	0 ± 0	0.217
	**34**	2 ± 4.14	5.07 ± 11.2	0 ± 0	0.281
	**44**	3 ± 5.28	4.62 ± 7.53	0 ± 0	0.212
**Itching**	**0**	1.33 ± 3.51	1.39 ± 3.01	0 ± 0	0.518
	**10**	0.67 ± 1.76	1.53 ± 3.75	0 ± 00	0.391
	**34**	1.33 ± 3.52	3.15 ± 8.31	0 ± 0	0.429
	**44**	2.2 ± 5.58	1.38 ± 3.01	0 ± 0	0.474

Visual analog scores (VAS) were expressed as means ± standard deviation, and *p* values were calculated using analysis of variance. No statistically significant differences in VAS results were observed between healthy volunteers and diabetic patients with or without diabetic peripheral neuropathy.

VAS, visual analog score; ANOVA, analysis of variance; HV, healthy volunteer; NDPN, diabetes mellitus without diabetic peripheral neuropathy; DPN, diabetic peripheral neuropathy.

### Visual analog scores

Although the patterns of brain activation were different between the three groups, the VAS results for both pain and itching were not significantly different (Table 3). There was no statistical difference in pain scores, but there were differences in central nucleus and cortical activation between the groups: this suggests that fMRI can detect the early stages of DPN-related CNS changes when the clinical symptoms are not obvious.

## Discussion

This study explored the differences in brain activity in response to thermal stimuli in diabetic patients with and without DPN using fMRI. The results suggest that, compared with NDPN patients and HV, DPN patients showed stronger activation not only in brain areas that participate in somatosensory pathways, but also in advanced cerebral areas that are in charge of cognitive processes.

### Correlation between function magnetic resonance imaging activation and thermal stimulus

#### Part 1. Activation pattern corresponding to pain sensation induced by thermal stimulus

In the interaction analysis, DPN patients showed stronger activation than NDPN patients or HV in cerebral areas that participate in somatosensory pathways of pain sensation (i.e., the right insular cortex, left caudate nucleus, frontal gyrus, and cingulate cortex; Figs 3 & 4A). This finding is consistent with previous studies, which have demonstrated that pain perception is a matrix composed of many interacting functional areas in the brain [17]. Augmented cerebral activities in the primary (S1) and secondary (S2) somatosensory cortices, lateral frontal lobe, cerebellum, anterior cingulum, and thalamus have been observed in DPN patients [7,13]. These brain areas are considered responsible for distinguishing the position and intensity of painful stimuli [7]. Besides, decreased gray matter volume in cerebral regions related to somatic sensation and significant shrinkage of the spinal cord have been documented in DPN and subclinical DPN patients [18,19]. However, in this study, despite activation of these pain-related cerebral areas, DPN patients did not experience significantly more pain than HV and NDPN participants (Table 3). There could be several reasons for this. First, the thalamus is regarded as the gateway through which pain sensation enters the brain; it amplifies pain messages transmitted from peripheral neurons [20]. Hypervascularity and hyperexcitability of the thalamus has been demonstrated in painful DPN, whereas hypovascularity and hypoexcitablity are features of painless DPN [21]. In our study, no thalamus activation was observed in DPN patients, which may explain why no significant pain sensation was reported. Second, nerve damage in DPN is irreversible and progressive [22]. Decreased ability to constrict blood vessels [23] and greater impairment of the nerve axon reflex mediated by C fibers in response to arousal stimuli [24] are more likely to be seen in painless DPN than in painful DPN. As nerve damage progresses, pain increases until the nerve is so severely damaged that pain signals are no longer transmitted to the CNS. The result is that pain sensation decreases and the feet begin to feel numb. This is consistent with our results, which show decreased VAS results in DPN patients compared with in NDPN patients, although without statistical significance. Third, although cerebral areas recognized to be part of the pain matrix were activated in patients with painless DPN in our study, it may be that the pattern, sequence, and strength of cerebral activation was different from that evident in painful DPN. Previous studies have shown that painful DPN responds more strongly to acute thermal stimulus than painless DPN [25]. The activation in these brain areas may be insufficient to produce pain in patients with painless DPN.

In addition to the cerebral areas involved in the somatosensory pathway, more advanced cognition-related areas, including the left hippocampus and left fusiform gyrus, were also activated in DPN patients in response to 10°C stimulation (Fig 4B). The hippocampus is not only responsible for memory and learning [26], but is also related to spatial orientation [27], whereas the fusiform gyrus, which is located in the visual cortex, has the function of face recognition [28]. Our study suggests that advanced cognitive abilities such as learning, memory, spatial orientation, and face recognition may be affected in DPN patients. This result, which has not been reported before, is a major finding of this study. A previous meta-analysis demonstrated cognitive decline in NDPN patients [29], and it has been suggested that dysregulation of glycemic control is responsible for this cognitive impairment [30]. Our study confirms the involvement of the CNS in DPN, and suggests that the changes in these cerebral areas may precede clinically evident cognitive decline. Moreover, although we only stimulated the right legs of the participants, both the right and left cingulum were activated (Fig 4C), implying interconnections between cingulate nerve fibers. This explains why some patients have bilateral symptoms although their EMG findings only indicated unilateral dysfunction.

On comparing the activation patterns between NDPN patients and HV in response to 0°C stimulation, we found that activation in cerebellar vermis 1–3 was more prominent in NDPN patients (Fig 2B). The cerebellar vermis is composed of the anterior lobe vermis, posterior lobe vermis, and posterolateral region of the posterior lobe vermis. The main function of the vermis is to process proprioceptive and exteroceptive afferent signals and modulate muscle tone [31]. Thus, NDPN patients without complaints of peripheral neuropathy may still have subclinical changes in these areas.

This study has some limitations. Overall, activation in the right temporal lobe, right insula, left caudate nucleus, and frontal gyrus was significantly greater in DPN patients than in HV (Fig 3). However, when the response to each different temperature stimulus was examined separately, there was no significant difference between DPN patients and HV. There are several possible explanations for this finding. For example, the statistical analysis was influenced by the threshold cluster size of > 20. It is possible that some significant differences were masked when cluster size was limited to > 20. Moreover, it is possible that we missed the most sensitive temperature point in DPN patients. Stimulation with more distinctive temperatures should be performed in future studies.

#### Part 2. Activation pattern corresponding to the itching sensation induced by thermal stimulus

Interaction analysis showed stronger activity in the cerebellar vermis and cingulum in NDPN and DPN patients than in HV (Figs 2 & 3). Both the cerebellar vermis and cingulum are involved in the recognition of an itching sensation [21,31]. Activation of the cerebellar vermis can lead to both pain and itching [31]. However, no patients in the DPN group reported itching in response to thermal stimulation. One reason for this could be that the temperature stimulus used in this experiment was insufficient to produce itching. Moreover, itching reflects a complicated network of brain activity. The hippocampus, amygdala, and subcortical cerebral areas are all involved in the itching network [32]. The activation of various other brain areas, such as the thalamus, anterior supplementary motor area, anterior insula, parietal inferior gyrus, and anterolateral prefrontal area, as well as the suppression of the orbitofrontal, medial prefrontal, medial cingulate, and primary motor cortex, are also related to the histamine-stimulated scratch response [33]. Therefore, activation of only the cerebellar vermis or cingulum may be insufficient to produce clinical itching. Because interactions exist between the peripheral and central pathways in the pathogenesis of DPN, it is necessary to explore whether peripheral neuropathy leads to activation of the above brain areas, or whether cortical activation results in peripheral neuropathic pain/itching. Although fMRI has many limitations and cannot always be performed in clinical settings because of its cost, it has been widely used to detect the neurological changes in pediatric epilepsy, Alzheimer’s disease, and other neurodegenerative diseases [34–36]. Our study demonstrates the utility of fMRI as a noninvasive and radiation-free tool for the diagnosis of DPN.

## Conclusions

In conclusion, screening with fMRI may be useful for the early detection of DPN-related CNS impairment. Our results indicate that CNS impairment related to diabetic neuropathy may not be limited to motion- and sensation-related cortical regions, but that cognition-associated cerebral regions such as the hippocampus and fusiform gyrus are also affected by the functional changes caused by DPN. This suggests that fMRI can detect the early stages of cognitive impairment in DPN patients before the symptoms become clinically significant.
